# Targeted PHA Microsphere-Loaded Triple-Drug System with Sustained Drug Release for Synergistic Chemotherapy and Gene Therapy

**DOI:** 10.3390/nano14201657

**Published:** 2024-10-16

**Authors:** Shuo Wang, Chao Zhang, Huandi Liu, Xueyu Fan, Shuangqing Fu, Wei Li, Honglei Zhang

**Affiliations:** 1 State Key Laboratory of New Pharmaceutical Preparations and Excipients, Key Laboratory of Medicinal Chemistry and Molecular Diagnosis of the Ministry of Education, Key Laboratory of Chemical Biology of Hebei Province, College of Chemistry and Materials Science, Hebei University, Baoding 071002, China; yingyang@stumail.hbu.edu.cn (S.W.); persistent_ghw@stumail.hbu.edu.cn (H.L.); 2024400355@buct.edu.cn (X.F.); fushuangqing@stumail.hbu.edu.cn (S.F.); 2 Department of Life Science, Hengshui University, Hengshui 053000, China; chaozhang@hsnc.edu.cn

**Keywords:** amphiphilic core shell, polyhydroxyalkanoates, chemogene drugs, synergetic therapy, sustained drug release, microparticles

## Abstract

The combination of paclitaxel (PTX) with other chemotherapy drugs (e.g., gemcitabine, GEM) or genetic drugs (e.g., siRNA) has been shown to enhance therapeutic efficacy against tumors, reduce individual drug dosages, and prevent drug resistance associated with single-drug treatments. However, the varying solubility of chemotherapy drugs and genetic drugs presents a challenge in co-delivering these agents. In this study, nanoparticles loaded with PTX were prepared using the biodegradable polymer material poly(3-hydroxybutyrate-co-3-hydroxyhexanoate) (PHBHHx). These nanoparticles were surface-modified with target proteins (Affibody molecules) and RALA cationic peptides to create core-shell structured microspheres with targeted and cationic functionalization. A three-drug co-delivery system (PTX@PHBHHx-ARP/siRNA_GEM_) were developed by electrostatically adsorbing siRNA chains containing GEM onto the microsphere surface. The encapsulation efficiency of PTX in the nanodrug was found to be 81.02%, with a drug loading of 5.09%. The chemogene adsorption capacity of siRNA_GEM_ was determined to be 97.3%. Morphological and size characterization of the nanodrug revealed that PTX@PHBHHx-ARP/siRNA_GEM_ is a rough-surfaced microsphere with a particle size of approximately 150 nm. This nanodrug exhibited targeting capabilities toward BT474 cells with HER2 overexpression while showing limited targeting ability toward MCF-7 cells with low HER2 expression. Results from the MTT assay demonstrated that PTX@PHBHHx-ARP/siRNA_GEM_ exhibits high cytotoxicity and excellent combination therapy efficacy compared to physically mixed PTX/GEM/siRNA. Additionally, Western blot analysis confirmed that siRNA-mediated reduction of Bcl-2 expression significantly enhanced cell apoptosis mediated by PTX or GEM in tumor cells, thereby increasing cell sensitivity to PTX and GEM. This study presents a novel targeted nanosystem for the co-delivery of chemotherapy drugs and genetic drugs.

## 1. Introduction

Malignant tumors, being a prominent cause of mortality, pose a significant threat to global public health, with a rising incidence rate annually [1]. Chemotherapy remains a prevalent approach in clinical oncology due to its simple and convenient treatment process. However, the development of drug resistance (DR) presents a substantial challenge to the efficacy of anti-cancer chemotherapeutic agents, impeding the optimal utilization of chemotherapy [2]. Recent studies have highlighted two primary strategies for reversing multi-drug resistance in tumors: the use of multi-drug combination chemotherapy and the synergistic application of chemotherapy drugs with inhibitors. In the context of the former strategy, the combined use of chemotherapeutic drugs with distinct molecular mechanisms can synergistically enhance anti-tumor effects, thereby augmenting tumor cell eradication while minimizing the risk of drug resistance and overlapping toxicities [3]. For instance, Lan et al. demonstrated that a combination of chemotherapy with paclitaxel (PTX), oxaliplatin, 5-fluorouracil, and leucovorin could effectively combat drug resistance and treat recurrent or metastatic gastric cancer [4]. Nonetheless, the effectiveness of multi-drug combination chemotherapy critically hinges on the precise molar ratios and meticulous sequential administration of the selected drugs [5].

The synergistic treatment strategy involves the use of inhibitors targeting drug resistance-associated proteins, primarily small molecule inhibitors [6] and therapeutic gene products (ASOs, siRNAs, and miRNAs) [7]. These products aim to restore the sensitivity of tumor cells to relevant chemotherapy drugs by precisely upregulating or downregulating specific gene expression. It should be noted that variations in the physicochemical properties of chemotherapeutic drugs and macromolecular gene therapeutics may result in differences in their biological distribution and tumor accumulation when administered individually. A recent trend in research, known as chemogene, focuses on the co-delivery of chemotherapy drugs and nucleic acids. This approach involves integrating drugs into corresponding nucleic acids, serving as both gene regulators and chemotherapy drugs [8]. Mou et al. [9] incorporated floxuridine (F) into antisense oligonucleotides and linked the chemogene with polycaprolactone to create spherical nucleic acids (SNAs) through self-assembly for the treatment of drug-resistant tumors. Zhu et al. [10] conjugated two PTX molecules with an F-integrated antisense oligonucleotide to produce a precise macromolecular drug-chemogene micelle, which exhibited a synergistic antitumor effect and significantly inhibited tumor growth. Simonenko developed a single drug molecule that co-delivered gemcitabine (GEM) and a synergistic siRNA simultaneously. The siRNA-GEM constructs showed a 5–30-fold increase in potency compared to GEM alone [11]. However, there is a need to enhance the coupling efficiency of hydrophobic side chains used in the formation of spherical nucleic acids. Additionally, addressing the inherent limitations of chemogene SNAs, such as premature disassembly, undesired liver accumulation, and lack of active targeting, poses a challenge [12]. Therefore, when designing chemogene-like nanoparticles, factors such as simplicity, high efficiency, stability, and active targeting capability should be considered.

The aforementioned limitations can be effectively addressed by co-delivery nanoplatforms, including cationic polymers, micelles, liposomes, and porous organic/inorganic nanomaterials. However, concerns persist regarding their drug-loading capacity, biosafety, biocompatibility, biodegradability, and costs, necessitating further improvement. Polyhydroxyalkanoates (PHAs) represent a diverse group of biopolyesters synthesized by various bacteria as intracellular carbon and energy storage materials [13]. Numerous studies have highlighted PHA as a promising biomedical material due to its varied and superior mechanical, biodegradable, and tissue-compatible properties [14]. PHA has been explored for applications in biological control agents, drug carriers, biodegradable implants, tissue engineering, and anticancer agents [15]. In our previous study, a novel amphiphilic cationic peptide-coated PHA nanosphere was developed, utilizing poly(3-hydroxybutyrate-co-3-hydroxyhexanoate) (PHBHHx) as the hydrophobic core and the RALA cationic peptide as the hydrophilic shell. This platform facilitated the co-delivery of hydrophobic drugs like curcumin (Cur) and nucleic acid drugs such as a 15-consecutive oligomeric 5-fluorodeoxyuridine strand (FUdR_15S_) [16]. Nevertheless, the lack of targeting in this nano-delivery system hinders its broader application.

This study aims to enhance the targeting capabilities of the PHBHHx-RALA cationic carrier by incorporating targeting ligands. Affibody, a small (6.5 kDa) affinity protein based on a three-helix bundle domain framework, is proposed as a suitable alternative to antibodies for targeted delivery [17]. High-affinity affibodies have been identified for various cancer-associated molecular targets [18]. However, the challenge lies in arranging Affibody on the surface of microspheres. The optimal combination of Affibody and RALA was selected and assembled with PHBHHx hydrophobic particles to create PHBHHx microspheres with a “core-shell” structure, enabling both targeting and cationic functionalization. These microspheres were employed for the co-delivery of chemotherapy drugs PTX and chemogene (siRNA chains embedded with GEM, siRNA_GEM_) to achieve synergistic treatment for breast cancer. Subsequently, the encapsulation efficiency, drug-loading capacity, and adsorption capacity of siRNA_GEM_ and PTX were individually determined. The size and morphology of the nanodrug were characterized, and its stability and release profile were assessed. The uptake of the nanodrug by breast cancer cells with varying HER2 expressions was studied, along with the intracellular localization of the nanodrug post-uptake. Furthermore, the toxicity of the nanodrug on HER2 high-expression cells (BT474) and HER2 low-expression cells (MCF-7) was evaluated, and the apoptotic mechanism induced by the nanodrug in vivo was investigated.

## 2. Materials and Methods

### 2.1. Materials

PTX and GEM were purchased from Shanghai Yuanye Bio-Technology Co., Ltd. The siRNA_GEM_ was modified by replacing cytosine with GEM nucleotides. The specific design is as follows: Bcl-2 sense 5-AGTA[GEM]AT[GEM][GEM]ATTATAAG[GEM]T-3′ and antisense 5-AG[GEM]TTATAATGGATGTA[GEM]T-3′ [19]. This siRNA was synthesized by Nanjing GenScript Biotechnology Co., Ltd. Fetal bovine serum, ribonuclease, trypsin cell digestion solution, DAPI staining solution, thiazole blue bromide tetrazolium, ECL luminous reagent, and penicillin/streptomycin solution (100X) were purchased from Sangon Biotech Co., Ltd. (Shanghai, China) Primary antibodies targeting Bcl-2 (bsm-33047M) and β-actin (bsm-33036M) were obtained from Bioss Antibodies Co., Ltd. (Beijing, China) Other commonly utilized reagents are available for commercial purchase.

### 2.2. Preparation of Targeted Microsphere-Loaded Triple-Drug System

PTX@PHBHHx nanoparticles were synthesized through an emulsion solvent evaporation technique [16]. Specifically, 1 mg of PTX and 50 mg of PHBHHx were dissolved in 7 mL of dichloromethane via ultrasonic dissolution. The resulting mixture was slowly added dropwise into 60 mL of 1% PVA solution, with the addition of 0.5 mL of Tween-20, followed by ultrasonic homogenization. Subsequently, residual dichloromethane was removed using rotary evaporation. The concentrated solution underwent centrifugation to collect the precipitate, which was then washed thrice with distilled water and subjected to lyophilization to yield PTX@PHBHHx nanoparticles. The PTX content in PTX@PHBHHx was quantified using high-performance liquid chromatography (HPLC) with an Agilent C-18 column (4.6 × 250 mm, 5.0 µm) [20]. The mobile phase consisted of a 4% glacial acetic acid solution (52%) and acetonitrile (48%) with a linear gradient flow rate of 1.0 mL/min, and UV detection at 425 nm. The encapsulation efficiency (EE) and loading content (LC) were determined using an external standard method to establish a standard curve, and calculated using the following formulas:

(1) $$\mathrm{EE}\;=\frac{W_{t}-\;C\;\times \;V}{W_{t}}\times \;100\%$$

(2) $$\mathrm{LC}\;=\frac{W_{t}-C\times V}{W_{s}}$$

W_t_ is the dosage of PTX; C is the concentration of the drug in the supernatant; V is the total volume of this batch of preparation, and W_s_ is the total mass of the drug-loaded microspheres.

The design and preparation of the fusion proteins were subsequently conducted. Two distinct sequences of fusion proteins, Affibody-RALA-PhaP (ARP) and PhaP-RALA-Affibody (PRA), were designed. The gene fragments of the fusion proteins, obtained using a one-step cloning kit, were digested with the endonucleases BamH I and EcoR I. Subsequently, they were inserted into the pCold I plasmid to obtain recombinant plasmids pCold I-Affibody-rala-phaP (pCold I-arp) and pCold I-phaP-rala-Affibody (pCold I-pra) (Figure S1). The authenticity of the recombinant plasmids was confirmed through DNA sequencing. The resultant positive plasmids were transformed into Escherichia coli BL21(DE3). These recombinant strains were cultured in LB medium, and upon reaching an optical density (OD) value of approximately 0.6, 0.5 mM/L IPTG was added to induce the expression of the recombinant proteins at a low temperature. Following induction, the bacterial culture was centrifuged at 6000 rpm, 4 °C for 15 min, washed three times with a PBS solution, and the bacterial precipitate was collected and subjected to ultrasonic disruption. The disrupted bacterial solution was centrifuged again at 13,000 rpm, 4 °C for 40 min to obtain the precipitate. Subsequently, 5 mL of protein-denaturing solution was added to the precipitation and stirred until dissolved. The solution was centrifuged at 13,000 rpm for 40 min, and the resulting supernatant was collected. The protein concentration was quantified using the BCA method and confirmed by 12% denatured polyacrylamide gel electrophoresis [21]. The collected protein was divided and stored at −80 °C for future use.

Finally, the PTX@PHBHHx composite was combined with high-expression fusion proteins. A total of 8 mg of PTX@PHBHHx microspheres were measured and introduced into 3 mL of ARP fusion protein solution (3 mg/mL). The assembly was performed at low speed at 4 °C overnight. Subsequently, the microspheres underwent centrifugation at 13,000 rpm for 15 min at 4 °C; the precipitates were collected as PTX@PHBHHx-ARP microspheres.

The microspheres were resuspended in 200 μL of pre-cooled PBS buffer (pH = 5.0), followed by the addition of 50 μL of siRNA_GEM_ (concentration of 20 mmol/L). The solution was gently stirred at low speed at 4 °C for 2 h. Subsequently, the solution underwent centrifugation at 13,000 rpm, 4 °C, for 20 min, resulting in the collection of the sediment as PTX@PHBHHx-ARP/siRNA_GEM_. The supernatant was analyzed using a fluorescence photometer, and the adsorption rate of the siRNA_GEM_ chain was determined using the formula below, where Wt represents the amount of siRNA_GEM_ added, and W is the amount of siRNA_GEM_ remaining in the supernatant after adsorption [22].

(3) $$\;\mathrm{Adsorption}\;\mathrm{rate}=\frac{W_{t}-\;W}{W_{t}}\times \;100\%$$

### 2.3. Characterization of Targeted Microsphere-Loaded Triple-Drug System

PTX@PHBHHx and PTX@PHBHHx-ARP/siRNA_GEM_ samples were diluted tenfold with PBS and transferred into a 5 mL colorimetric dish. The zeta potential was assessed using dynamic light scattering (DLS, Brookhaven Instruments Corporation, Holtsville, NY, USA). Subsequently, 10 µL aliquots of the samples were applied to a silicon wafer, air-dried overnight, and examined using scanning electron microscopy (SEM, JSM-7500F, Hitachi, Ltd., Tokyo, Japan). Additionally, samples were deposited on a 200-mesh copper grid, negatively stained with a 1% solution of phosphotungstic acid (pH 7.4), rinsed thrice with distilled water, and air-dried overnight for observation under a transmission electron microscope (TEM, H-600, Hitachi, Ltd., Tokyo, Japan). Infrared testing was performed using a Fourier infrared spectrometer (FTIR, Nicolet iS10, Thermo Fisher Scientific, Waltham, MA, USA), and the obtained data were analyzed using OMNIC 8.2 software. The presence of siRNA_GEM_ on the microspheres was visualized using confocal laser scanning microscopy (CLSM, Zeiss LSM 810, Oberkochen, Germany).

### 2.4. Stability Analysis of PTX@PHBHHx-ARP/siRNA_GEM_

The stability assessments of PTX@PHBHHx-ARP/siRNA_GEM_ were carried out under simulated in vivo conditions. A 50 μL solution of PTX@PHBHHx-ARP/siRNA_GEM_ was combined with an equal volume of 10% non-heat-inactivated fetal calf serum and then incubated at 37 °C for varying durations of 0.25, 0.5, 1, 2, 4, 8, 12, and 48 h. Furthermore, PTX@PHBHHx-ARP/siRNA_GEM_ was exposed to different pH buffers (acidic: pH = 4.5, neutral: pH = 7.4, and alkaline: pH = 9.0) at 37 °C for 2.0 h. Subsequent to this, PTX@PHBHHx-ARP/siRNA_GEM_ was treated with 0.36 U/mL DNase I for durations of 0.25, 0.5, 1, 2, 4, 8, 12, and 48 h. At various time points, 5 μL of the mixture was extracted, and equal volumes of a 1.5% heparin sodium solution were added, followed by 30 min incubation at 37 °C. Finally, the aforementioned mixtures were examined using 2% agarose gel and visualized with Tanon 2500 (Tanon Science and Technology Co., Ltd., Shanghai, China).

### 2.5. In Vitro Drug Release of PTX@PHBHHx-ARP/siRNA_GEM_

To investigate the in vitro release behavior of PTX and siRNA_GEM_ from PTX@PHBHHx-ARP/siRNA_GEM_, a 2 mL solution of PTX@PHBHHx-ARP/siRNA_GEM_ was introduced into a dialysis bag with a molecular weight cutoff (MWCO) of 10 kDa. The bag was then submerged in 10 mL of acetate buffer (pH 4.5) containing DNase II (20 U/mL) and subjected to continuous agitation at 100 rpm in a temperature-controlled incubator shaker (ZWYR-200D, LABWIT Scientific, Shanghai, China) set at 37 °C. At predetermined time intervals, 0.5 mL of release media was removed and replaced with 0.5 mL of fresh release media. The concentrations of PTX and siRNA_GEM_ in the solution were quantified using fluorescence spectrophotometry and high-performance liquid chromatography (HPLC).

### 2.6. Cell Cultures

Breast cancer cells BT474 and MCF-7 were acquired from the cell resource center at the Shanghai Biological Sciences Institute (Chinese Academy of Sciences, Shanghai, China). The cells were cultured in high-glucose DMEM supplemented with 10% FBS and 1% penicillin-streptomycin solution. The cells were then incubated at 37 °C in a humidified atmosphere with 5% CO_2_.

### 2.7. Cellular Uptake of PTX@PHBHHx-ARP/siRNA_GEM_

The uptake of PTX@PHBHHx-ARP/siRNA_GEM_ by cells was qualitatively analyzed by CLSM. BT474 and MCF-7 cells (1 × 10^5^ cells/well) were seeded separately in a laser confocal dish (NEST, Wuxi, China) and incubated overnight at 37 °C. Cells were treated with Cy5-labeled PTX@PHBHHx-ARP/siRNA_GEM_ for varying durations of 0.5, 1, 2, 4, 8, and 16 h. Following treatment, the culture medium was aspirated, and cells were washed three times with ice-cold PBS, fixed with 4% formaldehyde for 20 min at room temperature, and stained with 2.5 µg/mL DAPI for 30 min at 37 °C. After rinsing with PBS, 100 μL of 10% glycerol in aqueous solution was added, and the cells were examined using CLSM with a 40× oil objective.

The experiment on colocalization was conducted by utilizing a lysosome staining reagent to elucidate the mechanism of cellular uptake of PTX@PHBHHx-ARP/siRNA_GEM_. Following the incubation of BT474 and MCF-7 cells with PTX@PHBHHx-ARP/siRNA_GEM_ for 2 h, the lysosome was stained with Lyso-Tracker Green (50 nmol/L) for 0.5 h. To mitigate the issue of fluorescence quenching of lysosome green in cells, observations were promptly made post-incubation under dark conditions using laser confocal microscopy with a 40× oil objective.

### 2.8. Evaluation of the In Vitro Cytotoxicity of PTX@PHBHHx-ARP/siRNA_GEM_

In this study, in vitro cytotoxicity assays were conducted on cancer cells utilizing the MTT method. Briefly, BT474 and MCF-7 cells in the exponential growth phase were collected and seeded into 96-well plates at a density of 4 × 10^3^ cells/well. Following a 24 h incubation period at 37 °C, the culture medium was substituted with 100 µL of fresh medium containing varying concentrations of PTX, GEM, siRNA, PTX/GEM/siRNA, and PTX@PHBHHx-ARP/siRNA_GEM_. The cells were then further incubated for 48 h. Subsequently, 10 µL of MTT solution (5 mg/mL) was added to each well, and the plates were incubated at 37 °C for 4 h. The supernatant was aspirated, and 100 µL of DMSO was added to each well. The absorbance was measured at 490 nm. The results were presented as the mean of three independent experiments, with each experiment performed in quintuplicate. The dose-response curve was generated by calculating the percentage of cell viability using the following formula:

Cell viability (%) = [OD490 (Sample) − OD490 (Blank)]/[OD490 (Control) − OD490 (Blank)] × 100%

The inhibitory concentrations causing 50% growth inhibition (IC50 value) of PTX, GEM, and siRNA alone and in combination were determined using an online calculator (https://www.aatbio.com/tools/ic50-calculator, accessed on 8 September 2022).

The combination index (CI) of the three drugs was determined using the methodology outlined in the literature [23], The synergistic effect of PTX, GEM, and siRNA in PTX@PHBHHx-ARP/siRNA_GEM_ was assessed. CI values below 1, equal to 1, and above 1 indicate synergism, additivity, and antagonism, respectively.

### 2.9. Western Blot Analysis

BT474 and MCF-7 cells were seeded in six-well plates at a density of 2 × 10^5^ cells/well and incubated for 24 h. Subsequently, PTX, GEM, PTX/GEM/siRNA, and PTX@PHBHHx-ARP/siRNA_GEM_ were added to the plates at the same cell density, with final concentrations of 10 µM for PTX and 100 µM for GEM. After another 24 h, the culture media were aspirated, and the cells were washed twice with cold PBS buffer before being harvested. The cell pellets were lysed in RIPA buffer (0.5% NP-40, 50 mM Tris-HCl, 120 mM NaCl, 1 mM EDTA, 0.1 mM Na_3_VO_4_, 1 mM NaF, 1 mM PMSF, and 1 µg/mL leupeptin; pH 7.5), and the protein concentrations were determined using the BCA kit. Following SDS-polyacrylamide gel electrophoresis, the proteins were transferred to PVDF membranes, which were then blocked with 5% milk in TBST (5 mM Tris-HCl, 136 mM NaCl, and 0.1% Tween-20, pH 7.6) for 1 h. The membranes were probed with primary antibodies against Bcl-2 and β-actin overnight at 4 °C, washed three times with 1× TBST, incubated with horseradish peroxidase-conjugated secondary antibodies at room temperature for 1 h, and washed three times with 1× TBST. Protein bands were visualized using the Tanon 5200 ECL system (Tanon Science & Technology Co., Ltd., Shanghai, China) and analyzed with Image J 1.53k software.

### 2.10. Flow Cytometry Analysis

BT474 and MCF-7 cells were seeded in six-well plates at a density of 4 × 10^5^ cells/well and incubated for 24 h. Subsequently, PTX, GEM, PTX/GEM/siRNA, and PTX@PHBHHx-ARP/siRNA_GEM_ were added to the plates at the same cell density, with final concentrations of 10 µM for PTX and 100 µM for GEM. After another 24 h, the culture media were removed, and the cells were rinsed twice with cold PBS buffer for collection. The cells were then trypsinized at 2000 rpm for 5 min, washed thrice with PBS, and finally harvested. The cells were resuspended in 300 μL of 1× binding buffer as per the kit instructions. Subsequently, the cells were incubated with Annexin V-FITC at room temperature in the dark for 15 min using 5 µL. Prior to machine detection, the cells were stained with 5 µL of PI. Finally, 200 μL of the binding buffer solution was added, and the cell apoptosis was assessed using flow cytometry.

### 2.11. Statistical Analysis

All samples were prepared and tested at least thrice. Data were presented as mean ± standard deviation. Significant differences among groups were determined using Newman–Keuls analysis. Differences were considered significant at * *p* < 0.05, highly significant at ** *p* < 0.01, and extremely significant for *** *p* < 0.001.

## 3. Results and Discussion

### 3.1. Preparation of Targeted Microsphere-Loaded Triple-Drug System

**Scheme 1:**
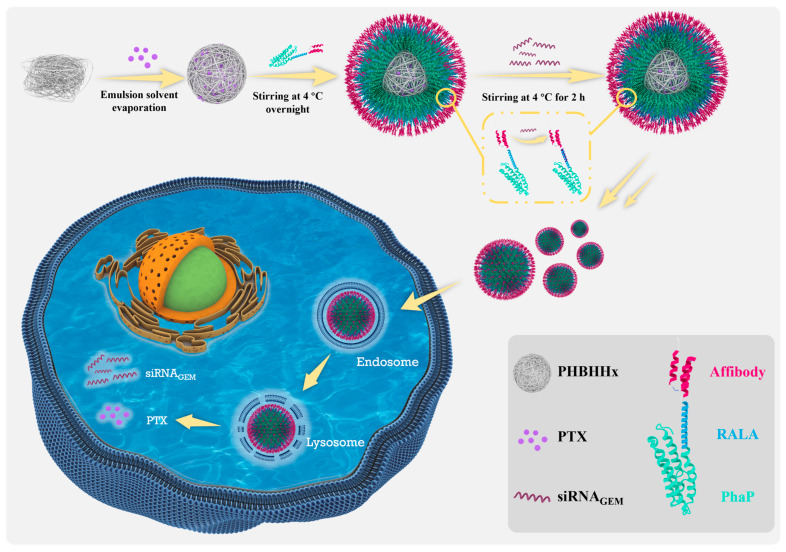
The assembly process of PTX@PHBHHx-ARP/siRNA_GEM_ and their synergistic cancer therapy.

The co-delivery system for novel chemotherapy drugs and gene drugs has been developed, as illustrated in Scheme 1. Initially, the chemotherapy drug PTX was enclosed within PHA particles through the solvent evaporation technique to form the hydrophobic core of PTX@PHBHHx. The concentration of PTX in PTX@PHBHHx post-assembly was determined using the external standard method. At a PTX to PHBHHx ratio of 1:50, the encapsulation efficiency of PTX was found to be 81.02%, with a drug-loading capacity of 50.9 mg/g.

A hydrophilic shell was constructed with HER2 targeting and cation functionalization. Genes encoding a fusion protein of Affibody and the RALA cationic peptide, connected to the N-terminus and C-terminus of the “PhaP” protein, were designed and synthesized. These two genes were ligated with the pCold I plasmid and transformed into *E. coli* BL21(DE3) competent cells. Upon comparing the expression of the fusion proteins induced by the two recombinant strains, it was observed that the expression of the Affibody-RALA-PhaP protein was higher. Consequently, *E. coli* BL21(DE3)/pCold I -*arp* was chosen as the target strain. The fusion protein ARP was purified using affinity chromatography, and its molecular weight was determined to be 34 kDa (Figure S2). The hydrophobic core of PTX@PHBHHx and the targeting cationic hydrophilic shell of ARP self-assembled into a nanocomplex of PTX@PHBHHx-ARP through strong hydrophilic and hydrophobic interactions. To confirm the attachment of the ARP protein to the surface of PTX@PHBHHx particles, the assembled nanocomplex was directly subjected to SDS-PAGE. The fusion protein ARP, with a molecular weight of 34 kDa, was found to bind to the surface of the nanoparticles (Figure S3a).

The nanocomplex and the negatively charged heterozygous siRNA_GEM_ were combined to create three-drug co-delivery microspheres of PTX@PHBHHx-ARP/siRNA_GEM_ through electrostatic interaction. The loading capacity of siRNA_GEM_ on the nanocomplex was determined by measuring the amount of adsorbed siRNA_GEM_ using a fluorescence photometer. Based on the standard curve of siRNA_GEM_, it was found that 486.3 nmol of siRNA_GEM_ could be adsorbed by 1 mg of nanocomplex. Subsequently, imaging verification of siRNA_GEM_ chains modified with Cy5 on microspheres was conducted using laser confocal microscopy (Figure S3b). The results demonstrated that siRNA_GEM_ with red fluorescence was evenly adsorbed on the surface of PTX@PHBHHx-ARP, providing conclusive evidence that PTX@PHBHHx-ARP/siRNA_GEM_ was successfully assembled.

### 3.2. Characterization of Targeted Microsphere-Loaded Triple-Drug System

**Figure 1:**
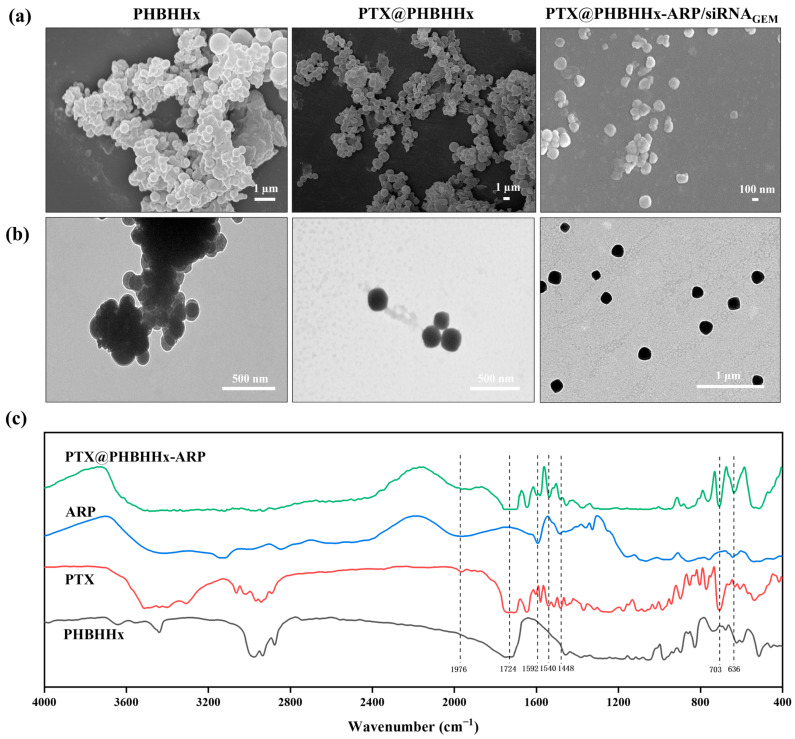
SEM (**a**), TEM (**b**), and FTIR (**c**) analysis.

The nanotopography of PHBHHx, PTX@PHBHHx, and PTX@PHBHHx-ARP/siRNA_GEM_ prepared via the solvent evaporation method was analyzed using SEM and TEM. The SEM image indicated that the PHBHHx particles were approximately 100 nm in size, displaying a smooth and spherical surface. Upon loading ARP protein and siRNA_GEM_, a slight increase in size was observed, and the surface of the microspheres appeared non-smooth (Figure 1a). TEM results also showed significant agglomeration of the PHBHHx and PTX@PHBHHx (Figure 1b), possibly due to the absence of loaded protein. Conversely, microspheres loaded with protein exhibited enhanced aggregation and improved dispersion. Furthermore, the zeta potential of the microspheres was determined, revealing that the surface potential of PTX@PHBHHx-ARP was +2.49 mA. This potential enables the adsorption of negatively charged siRNA_GEM_ chains through electrostatic interactions, facilitating the immobilization of siRNA_GEM_ on the protein surface of the microspheres.

In addition, infrared spectroscopy is further utilized to detect drug assembly. As shown in Figure 1c, the infrared characteristic peak of PHBHHx appears at 1724 cm^−1^, indicating a C=O stretching vibration, which is a significant feature of the polyester structure [24]. The infrared characteristic peak of paclitaxel corresponds to the C=C stretching vibration of the aromatic ring at 1540 cm^−1^, while the peak at 1448 cm^−1^ represents the bending vibration of the methyl group (-CH₃). Additionally, it also exhibits a stretching vibration of C=O or -C=O at 1724 cm^−1^ [25]. ARP, a protein, displays an amide II band at 1592 cm^−1^, which is associated with N-H bending and C-N stretching vibrations [26]. The assembled composite of PTX@PHBHHx-ARP contains characteristic infrared peaks from these individual components. The absence of peaks corresponding to new functional groups indicates that only secondary bonds were involved in the assembly process of our composite, with no covalent bonding occurring. PTX is encapsulated within PHBHHx particles through hydrophobic interactions, similar to how Zein protein can encapsulate various insoluble compounds [27]. The ARP protein layer adheres to the surface of PTX@PHBHHx through hydrophobic-hydrophilic interactions. Finally, the siRNA strand binds to the protein layer of the microspheres via electrostatic adsorption.

### 3.3. Stability Analysis of PTX@PHBHHx-ARP/siRNA_GEM_

The stability of PTX@PHBHHx-ARP/siRNA_GEM_ was evaluated by incubating the system in a serum solution and various pH buffers. Figure S4a illustrates that the bands of PTX@PHBHHx-ARP/siRNA_GEM_, without heparin sodium treatment, are concentrated at the sample wells with a dark black color, indicating the sustained stability of the microsphere structure in serum. Upon treatment with heparin sodium, the agarose gel reveals the siRNA_GEM_ chains loaded on the microspheres, indicating no significant changes in PTX@PHBHHx-ARP/siRNA_GEM_ within 0–2 h in serum (Figure S4b). However, degradation of siRNA_GEM_ begins at 4–8 h, suggesting that PTX@PHBHHx-ARP/siRNA_GEM_ maintains good stability in serum, showing minimal impact from serum albumin within 0–8 h, with stability persisting up to 48 h without complete degradation. Following a 2 h incubation at various pH levels, as depicted in Figure S4c, siRNA_GEM_ exhibits robust stability in neutral (pH 7.2) and alkaline (pH 8.0) environments but poor stability in acidic (pH 4.5) conditions. This observation further supports the high stability of PTX@PHBHHx-ARP/siRNA_GEM_ in the system under neutral pH conditions. This stability is advantageous as it prevents the degradation of drugs before they enter cells and enhances drug release in the acidic environment of lysosomes [28].

The presence of the RNase I enzyme in the lysosomes of cancer cells enables the degradation of siRNA. To assess the stability of PTX@PHBHHx-ARP/siRNA_GEM_ in the presence of RNase I, the degradation process of RNase I on PTX@PHBHHx-ARP/siRNA_GEM_ was investigated in vitro to simulate the lysosomal environment of cells. PTX@PHBHHx-ARP/siRNA_GEM_ and RNase I were incubated at 37 °C, pH 4.5, for various durations and subjected to electrophoresis on a 2% agarose gel. The findings, as depicted in Figure S4d, demonstrate that PTX@PHBHHx-ARP/siRNA_GEM_ exhibits notable stability in the presence of RNase I, showing no degradation within 24 h, although the microsphere structure begins to deteriorate after 48 h. This suggests that drugs can be released upon exposure to lysosomes after cellular uptake, facilitating drug penetration into the nucleus and subsequent therapeutic effects.

### 3.4. In Vitro Drug Release of PTX@PHBHHx-ARP/siRNA_GEM_

**Figure 2:**
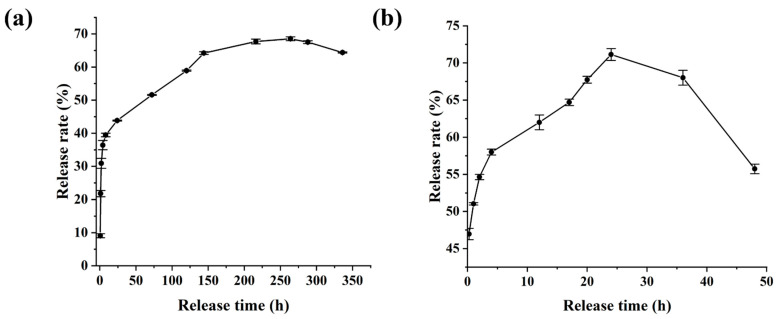
Release curves of PTX and siRNA_GEM_ in PTX@PHBHHx-ARP/siRNA_GEM_. (**a**) The PTX drug release curve. (**b**) The release curve of siRNA_GEM_.

The release kinetics of PTX and siRNA_GEM_ from PTX@PHBHHx-ARP/siRNA_GEM_ were further investigated in an acidic environment mimicking lysosomes (pH 4.5) through in vitro simulation using the dialysis method. As depicted in Figure 2a, the release of PTX reached 43.8% at 24 h, 51.6% at 72 h, and 58.9% at 120 h. Over time, there was a slight decrease in the rate of PTX release, with approximately 64.2% released at around 144 h and about 68.5% released at 264 h. The release profile tended to plateau, suggesting that PTX achieved maximum release. It is plausible that PHBHHx nanoparticles facilitate sustained PTX release. There have been studies utilizing poly(3-hydroxybutyrate) (PHB) microspheres loaded with paclitaxel (PTX) to investigate mouse liver cancer cells. The findings revealed that PHB particles released PTX uniformly over a period of two months, suggesting that PHB enhances the therapeutic effect of the anti-cancer drug [29]. Additionally, drugs loaded in PHBHHx, which shares similar properties with PHB, also demonstrate excellent extended-release effects in animal models [30]. In addition, the kinetics of PTX release from PTX@PHBHHx-ARP/siRNA_GEM_ was studied under neutral conditions (pH = 7.4). As shown in Figure S5, PTX exhibited a certain degree of release within 0–8 h, reaching a maximum release of 10% after 12 h. The reason for this phenomenon may be due to the diffusion movement of the partially encapsulated PTX on the surface of the microsphere, which exhibits a certain amount of drug release. After that, the release amount does not increase, indicating that the PTX encapsulated inside the microsphere can exist relatively stable under neutral conditions.

As illustrated in Figure 2b, the initial release of siRNA_GEM_ was around 51% after 1 h, followed by gradual release over time. By the 24 h mark, the release peak was observed at 71%. However, the release percentage decreased to 55.7% at 48 h. This decline could be attributed to the release of siRNA_GEM_ from the nanodrug, coupled with the loss of protection from the nanocarrier, which is eventually degraded by enzymes in the solution, leading to a reduction in the release rate.

### 3.5. Cellular Uptake of PTX@PHBHHx-ARP/siRNA_GEM_

**Figure 3:**
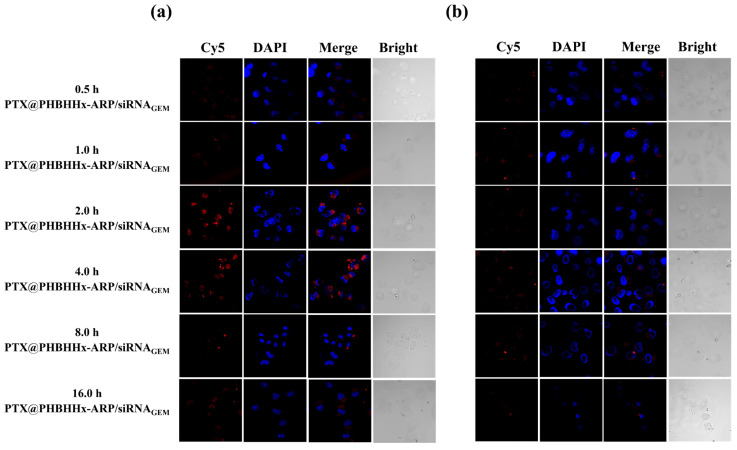
Drug uptake by BT474 and MCF-7 cells. (**a**) CLSM analysis of BT474 cells treated with PTX@PHBHHx-ARP/siRNA_GEM_ for different times. Panel (**b**) CLSM analysis of MCF-7 cells treated with PTX@PHBHHx-ARP/siRNA_GEM_ for different times. Scale bar: 20 μm.

To investigate the cellular uptake of PTX@PHBHHx-ARP/siRNA_GEM_, CLSM was employed to observe the uptake of the nanodrug in breast cancer BT474 and MCF-7 cells (Figure 3). The siRNA_GEM_ component of PTX@PHBHHx-ARP/siRNA_GEM_ was labeled Cy5. Analysis of the red fluorescence intensity in BT474 cells revealed that at 0.5 h, the intensity was low due to limited uptake of PTX@PHBHHx-ARP/siRNA_GEM_ by the cells. Over time, the red fluorescence intensity gradually increased. By 1 h, some PTX@PHBHHx-ARP/siRNA_GEM_ uptake was evident. Within the acidic lysosomal environment, drug release occurred, leading to enhanced fluorescence intensity, though not at its peak. By 2 h, the fluorescence intensity reached its maximum, indicating substantial cellular uptake of PTX@PHBHHx-ARP/siRNA_GEM_ and release from lysosomes. Subsequently, the fluorescence intensity decreased as uptake time prolonged, suggesting drug metabolism had commenced. At 16 h, the fluorescence intensity was diminished. Prolonged incubation time demonstrated a pattern of increasing and then decreasing nanodrug uptake in BT474 cells, indicating that the cells treated with PTX@PHBHHx-ARP/siRNA_GEM_ undergo uptake, absorption, and metabolism. Conversely, MCF-7 cells did not show significant changes in intracellular drug fluorescence intensity over time. This indicates that PTX@PHBHHx-ARP/siRNA_GEM_, which targets cells with high HER2 expression, exhibits low uptake in cells with low HER2 expression.

In order to assess the targeting efficiency of PTX@PHBHHx-ARP/siRNA_GEM_ with Affibody on breast cancer cells exhibiting varying levels of HER2 expression, the grayscale method was utilized to analyze laser confocal images of the two cell types. The uptake of PTX@PHBHHx-ARP/siRNA_GEM_ by the two cell types at different time intervals was plotted. As shown in Figure S6, there was no significant variance in the absorption of PTX@PHBHHx-ARP/siRNA_GEM_ between the HER2 high-expression cell line BT474 and the low-expression cell line MCF-7 at 0.5 h post-drug uptake. However, at 1 h, a notable difference was observed between the two groups, with BT474 cells exhibiting 1.4 times higher uptake of PTX@PHBHHx-ARP/siRNA_GEM_ compared to MCF-7 cells. By the 2 h mark, the uptake by BT474 cells was 4.2 times greater than that of MCF-7 cells. These results strongly indicated the effective targeting of the HER2 highly expressed protein on breast cancer cells’ surface by the Affibody. This specificity facilitated enhanced cellular penetration of the drug, thereby improving cancer treatment outcomes. Subsequent to 4 h, the metabolism of PTX@PHBHHx-ARP/siRNA_GEM_ by the cells resulted in a decrease in gray value over time. The substantial uptake of PTX@PHBHHx-ARP/siRNA_GEM_ by the HER2 high-expressing breast cancer cell line BT474 compared to MCF-7 cells underscored the targeting capability of the nanodrug developed in this study toward cells with elevated HER2 expression levels.

The localization of PTX@PHBHHx-ARP/siRNA_GEM_ in breast cancer cells was examined using CLSM. Upon staining with Lyso Tracker Green, lysosomes emitted green light, while the red fluorescence from PTX@PHBHHx-ARP/siRNA_GEM_ was observed to be localized within the lysosomes, as illustrated in Figure S7. The degree of co-localization between the red fluorescence of PTX@PHBHHx-ARP/siRNA_GEM_ and the green fluorescence of lysosomes was assessed by calculating Pearson’s correlation coefficient (R) using ZEN 2012 software, as shown in Figure S8. The overlapping curves of Cy5-containing drugs and lysosomes in Figure S9 suggested the entry of the drug into the lysosomes. The R-value of Cy5-siRNA_GEM_ in the lysosomes of BT474 cells was determined to be 0.91, while in MCF-7 cells, it was 0.80. These results indicated that the majority of PTX@PHBHHx-ARP/siRNA_GEM_ entered the intracellular lysosomes through endocytosis. From the results presented, a potential pathway for drug uptake and metabolism by cells can be inferred. The Affibody on PTX@PHBHHx-ARP/siRNA_GEM_ specifically targeted and bound to the HER2 receptor on the surface of breast cancer cells, facilitating the accumulation of PTX@PHBHHx-ARP/siRNA_GEM_ within these cancer cells. Furthermore, PTX@PHBHHx-ARP/siRNA_GEM_ entered the cytoplasm by either fusing with cell membranes or through endocytosis, subsequently being digested in lysosomes under the action of RNase I. PTX and siRNA_GEM_ interacted with the DNA of cancer cells, impairing the transcription and translation processes, ultimately leading to apoptosis [31].

### 3.6. Evaluation of the In Vitro Cytotoxicity of PTX@PHBHHx-ARP/siRNA_GEM_

**Figure 4:**
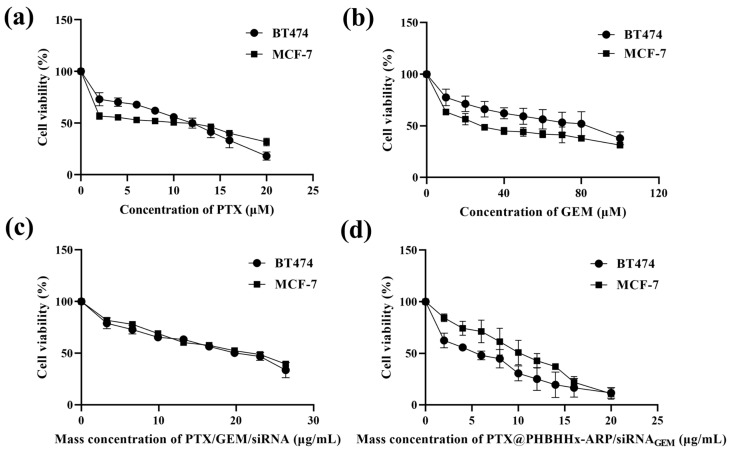
Survival of BT474 and MCF-7 cells treated with PTX, GEM, physical mixture of PTX/GEM/siRNA and PTX@PHBHHx-ARP/siRNA_GEM_. (**a**) The survival rates of the two cell types after 48 h incubation with PTX monotherapy. (**b**) The survival rates of the two cell types after 48 h incubation with GEM monotherapy. (**c**) The survival rates of the two cell types after 48 h of incubation with the physical mixture of PTX/GEM/siRNA. (**d**) The survival rates of the two cell types after 48 h incubation with PTX@PHBHHx-ARP/siRNA_GEM_.

To assess the anti-tumor efficacy of PTX@PHBHHx-ARP/siRNA_GEM_ on breast cancer cell lines BT474 and MCF-7, an MTT assay was performed to evaluate its impact on the cells. Both cell types were exposed to varying concentrations of free PTX, GEM, siRNA, a physical mixture of PTX/GEM/siRNA, and a nanodrug of PTX@PHBHHx-ARP/siRNA_GEM_ for 48 h. The results depicted in Figure 4 indicate a correlation between cell toxicity and drug concentration, with toxicity levels escalating as concentrations increase. The PTX single-drug group exhibited a gradual decrease in cell survival rates within the 0–20 μM range, mirroring the trend observed in the GEM monotherapy group where cell survival rates decreased as GEM concentrations increased within the 0–120 μM range. A comparison of the efficacy of free drugs versus the combined treatment of PTX, GEM, and siRNA revealed that the three-drug combination displayed a more potent anti-tumor effect. Furthermore, the nanodrug PTX@PHBHHx-ARP/siRNA_GEM_ exhibited higher toxicity levels compared to the physical mixture of PTX/GEM/siRNA. However, siRNA alone did not demonstrate any anti-tumor effect (Figure S10a). This lack of efficacy could be attributed to the negatively charged nature of siRNA, a double-stranded RNA that poses challenges for cancer cell uptake, thereby limiting its effectiveness. Furthermore, the cytotoxicity of siRNA_GEM_ was assessed by PHBHHx-ARP/siRNA_GEM_, and the results are shown in Figure S10b. Because PHBHHx-ARP had almost no toxicity to cells, the cytotoxicity exhibited came from the siRNA_GEM_ chain. This indicated that the siRNA_GEM_ chain had certain toxicity to both types of cells, and there was a correlation between toxicity and drug concentration. As the drug concentration increased, the toxicity level gradually increased. But compared to PTX@PHBHHx-ARP/siRNA_GEM_, the cytotoxicity of PHBHHx-ARP/siRNA_GEM_ was still relatively weak.

Furthermore, as illustrated in Table 1, both the physical mixture group and the nanodrug group demonstrated decreased IC50 values in BT474 and MCF-7 cells in comparison to the free drug. Notably, the nanodrug formulation of PTX@PHBHHx-ARP/siRNA_GEM_ exhibited the most significant reduction in IC50 values among all experimental groups. Nevertheless, free drugs or physical mixtures of drugs lack specificity in targeting, resulting in insignificant variations in their IC50 values when interacting with cells expressing high or low levels of the target. Within the nanodrug group, it is apparent that the IC50 values of PTX, GEM, and siRNA_GEM_ for BT474 cells (3.63 ± 1.4 nM, 1.49 ± 0.4 nM, and 30.90 ± 5.55 nM) were lower than those for MCF-7 cells (6.11 ± 1.8 nM, 2.53 ± 0.6 nM, and 43.75 ± 8.7 nM). These findings further corroborate the assertion that PTX@PHBHHx-ARP/siRNA_GEM_ enhanced the antitumor efficacy, with the Affibody structure augmenting the targeting specificity of the nanodrug [32].

The synergistic therapeutic effect of PTX, GEM, and siRNA was assessed in this study. The combination index (CI) of the three drugs was computed to evaluate the impact of the two distinct chemotherapy drugs on the cells. The results presented in Table S1 demonstrate that in BT474 cells, the CI value of the PTX@PHBHHx-ARP/siRNA_GEM_ treatment group (0.0007) was significantly lower compared to the three-drug physical mixture group (0.059). Similarly, in MCF-7 cells, the CI value of the PTX@PHBHHx-ARP/siRNA_GEM_ treatment group (0.0014) was notably lower than that of the physical mixture group (0.150). It demonstrated a remarkable synergistic anticancer effect of PTX, GEM, and siRNA in PTX@PHBHHx-ARP/siRNA_GEM_, particularly evident in cells with high HER2 expression. These results were consistent with those of combined administration of PTX and Bcl-2 siRNA [33], as well as PTX and GEM [34] in other cancer therapies. This outcome may be attributed to the increased sensitivity of cancer cells to PTX, caused by siRNA and GEM.

### 3.7. Western Blot Analysis

**Figure 5:**
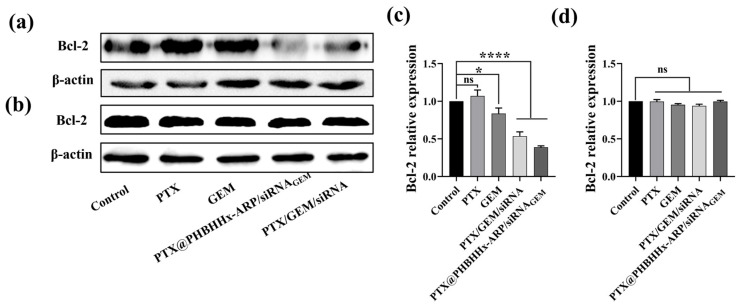
Western blot of Bcl-2 in (**a**) BT474 cells and (**b**) MCF-7 cells treated with free PTX, GEM, PTX/GEM/siRNA, and PTX@PHBHHx-ARP/siRNA_GEM_, respectively. (**c**) Significance analysis of Bcl-2 in BT474 cells. (**d**) Significance analysis of Bcl-2 in MCF-7 cells. Statistical analysis: * *p* < 0.05, **** *p* < 0.0001 and ns (No significant difference).

Researchers have identified Bcl-2 as a key regulator in the modulation of apoptosis and cell survival in response to various apoptotic triggers [35]. Elevated levels of Bcl-2 have been linked to the development of resistance to chemotherapy [36]. Consequently, siRNA_GEM_ is utilized to suppress the expression of the Bcl-2 gene, thereby augmenting cell sensitivity to PTX and GEM. The validation of Bcl-2 protein expression was conducted through Western blotting. Control groups included free PTX, GEM, and a physically combined PTX/GEM/siRNA. The outcomes are depicted in Figure 5. In BT474 cells, the changes in Bcl-2 proteins induced by PTX@PHBHHx-ARP/siRNA_GEM_ were notably more pronounced compared to the control group, resulting in downregulation. Conversely, in MCF-7 cells, the expression of Bcl-2 remained relatively stable following treatment with the same drug concentration. These findings suggest that Bcl-2 protein levels can be effectively reduced by Bcl-2 siRNA in HER2 overexpressing cells delivered via PHA-targeted microspheres. When combined with the cytotoxicity data, it further suggests that systems incorporating siRNA are more cytotoxic to cancer cells. This observation aligns with existing literature, which indicates that diminishing Bcl-2 expression significantly boosts cell apoptosis induced by PTX or GEM in tumor cells, thereby enhancing cell sensitivity to PTX [37] and GEM [38].

### 3.8. Flow Cytometry Analysis

**Figure 6:**
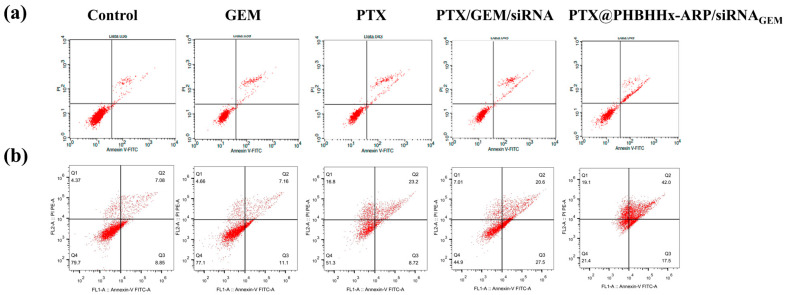
Scatter plots of apoptosis in (**a**) MCF-7 cells and (**b**) BT474 cells treated with free PTX, GEM, PTX/GEM/siRNA, and PTX@PHBHHx-ARP/siRNA_GEM_, respectively.

Flow cytometry was conducted to assess apoptosis in BT474 and MCF-7 cells utilizing the Annexin V-FITC/PI assay kit for detection. The cells were exposed to PTX, GEM, PTX/GEM/siRNA, and PTX@PHBHHx-ARP/siRNA_GEM_, and cell apoptosis was evaluated after 48 h. According to the findings of MCF-7 cell apoptosis in Figure 6a, in comparison to the untreated control group, the apoptosis rate was around 10% for the PTX group, 13% for the GEM group, and approximately 15% for the PTX/GEM/siRNA group. This suggests that the co-administration of drugs led to a slight increase in apoptosis compared to individual drug administration. Conversely, the apoptosis rate of cells treated with PTX@PHBHHx-ARP/siRNA_GEM_ was about 25%, indicating only a marginal rise compared to the other groups. As illustrated in Figure 6b, in the apoptosis of BT474 cells, the co-administration group exhibited higher toxicity compared to the individual drug group. The apoptosis rate reached about 50% in the co-administration group, while cells treated with PTX@PHBHHx-ARP/siRNA_GEM_ showed an apoptosis rate of approximately 80%. This suggests that the drug delivery system possesses structural integrity and effective targeted delivery properties, thereby reducing drug degradation during transit and facilitating targeted delivery to the tumor site, thus demonstrating the efficacy of the three-drug combination therapy.

## 4. Conclusions

This article presents a study on the development and synthesis of a fusion protein shell with both targeting and cationic functionalization. The fusion protein shell was created by combining the particle-binding protein PhaP with the target protein Affibody and RALA cationic peptide. The hydrophilic nature of the shell facilitates strong interactions with the hydrophobic core (PTX@PHBHHx) through hydrophilic–hydrophobic interactions, leading to the self-assembly of microsphere carriers, PTX@PHBHHx-ARP. Subsequently, the microspheres were hybridized with negatively charged siRNA_GEM_ chains through hydrogen bonding, resulting in the adsorption of siRNA_GEM_ onto the surface of the microspheres. This process culminated in the assembly of a three-drug co-delivery nanodrug, PTX@PHBHHx-ARP/siRNA_GEM_. The nano-microsphere exhibited high encapsulation efficiency for chemotherapy drugs (PTX) and a high loading capacity for chemogene drugs (siRNA_GEM_). Furthermore, the microspheres co-loaded with the three drugs demonstrated a smaller overall size and a more regular morphology. The nanodrugs exhibited good stability in simulated serum and neutral pH environments in vitro, maintaining their structure without damage. The targeting ability of the nanodrug was demonstrated by its effective targeting of BT474 cells with HER2 overexpression, while its targeting of MCF-7 cells with low HER2 expression was less effective. The nanodrug PTX@PHBHHx-ARP/siRNA_GEM_ also displayed high cytotoxicity and excellent combination therapy effects, leading to the downregulation of intracellular Bcl-2 protein and increased cell sensitivity to PTX and GEM. This study introduces a novel nano-delivery system for the co-delivery of chemotherapy drugs and genetic drugs.

## Figures and Tables

**Table 1:** IC50 values of different cells treated with different drugs.

Cell Line	IC50							
	PTX (μM)	GEM (μM)	PTX/GEM/siRNA (1:9:170)			PTX@PHBHHx-ARP/siRNA_GEM_		
			PTX (nM)	GEM (nM)	siRNA (μM)	PTX (nM)	GEM (nM)	siRNA_GEM_ (nM)
BT474	11.58 ± 0.6	82.95 ± 11.8	61.10 ± 2.1	21.9 ± 18.9	4.45 ± 0.3	3.63 ± 1.4	1.49 ± 0.4	30.90 ± 5.55
MCF-7	10.44 ± 0.8	46.44 ± 5.8	58.0 ± 7.3	23.51 ± 13.4	4.60 ± 0.9	6.11 ± 1.8	2.53 ± 0.6	43.75 ± 8.7

## Data Availability

The original contributions presented in the study are included in the article/supplementary material, further inquiries can be directed to the corresponding author/s.

## Supplementary Materials

The following supporting information can be downloaded at https://www.mdpi.com/article/10.3390/nano14201657/s1, Figure S1: Expression plasmids containing two fusion protein genes of different orders. (a) pCold I-*Affibody*-*rala*-*pha*P (pCold I-*arp*). (b) pCold I-*pha*P-*rala*-*Affibody* (pCold I-*pra*). Figure S2: (a) Analysis of PRA protein by SDS-PAGE gel electrophoresis. (M: Marker; Lane 1: before IPTG induction; Lane 2: after IPTG induction; Lane 3: supernatant after crushing; Lane 4: bacterial precipitation after crushing). (b) Analysis of ARP proteins by SDS-PAGE gel electrophoresis. (Lane M: Marker; Lane 1: supernatant of crushed cells; Lane 2: after induction of ARP protein; Lane 3: before induction of ARP protein; Lane 4: bacteria precipitate after fragmentation). Figure S3: Verification of the correctly assembled triple-drug microspheres. (a) SDS analysis of the ARP protein was attached to the surface of the microspheres. (b) Confocal imaging validation of siRNA_GEM_ was absorbed by microspheres. Figure S4: Analysis of the enzymatic release of PTX@PHBHHx-ARP/siRNA_GEM_ by agarose gel electrophoresis. (a) The stability of PTX@PHBHHx-ARP/siRNA_GEM_ at different times in 10% serum without heparin sodium treatment. (b) The stability of siRNA_GEM_ on PTX@PHBHHx-ARP/siRNA_GEM_ at different times in 10% serum with heparin sodium treatment. (c) The stability of PTX@PHBHHx-ARP/siRNA_GEM_ at different pH. (d) The stability of PTX@PHBHHx-ARP/siRNA_GEM_ in RNase I. Figure S5: Kinetics of PTX release under neutral conditions. Figure S6: Grayscale control of uptake of PTX@PHBHx-ARP/siRNA_GEM_ by two different cells. Figure S7: Intracellular localization map of the drug after uptake by BT474 (a) and MCF-7 (b) cells. After the cells were incubated with the drug for 2 h, the position of PTX@PHBHHx-ARP/siRNA_GEM_ after its entry into the cells was observed by CLSM. Scale bar: 20 μm. Figure S8: The co-localization of PTX@PHBHx-ARP/siRNA_GEM_ in lysosomes was analyzed by software. (a) The co-localization of PTX@PHBHx-ARP/siRNA_GEM_ in MCF-7 cells. (b) The co-localization of PTX@PHBHx-ARP/siRNA_GEM_ in BT474 cells. Figure S9: Localization analysis of PTX@PHBHx-ARP/siRNA_GEM_ in lysosomes in both cell types. (a) Lysosomal co-localization in BT474 cells. (b) Lysosomal co-localization in MCF-7 cells. Figure S10: The survival rates of the two cell lines were observed after 48 h of treatment. (a) siRNA. (b) PHBHHx-ARP/siRNA_GEM_. Table S1: CI values of different cells treated with three different combinations of drugs.
