# Supplementary material for: Targeted PHA Microsphere-Loaded Triple-Drug System with Sustained Drug Release for Synergistic Chemotherapy and Gene Therapy
Source: Nanomaterials (Basel). 2024 Oct 16;14(20):1657. doi: 10.3390/nano14201657 (PMC11510473; doi:10.3390/nano14201657)
Supplement: Supplementary file 1 [file nanomaterials-14-01657-s001.zip › nanomaterials-3219796-supplementary.pdf]

nanomaterials

MDPI

Article

Targeted PHA Microsphere-Loaded Triple-Drug System with Sustained Drug Release for Synergistic Chemotherapy and Gene Therapy

Shuo Wang [1], Chao Zhang [2], Huandi Liu [1], Xueyu Fan [1], Shuangqing Fu [1], Wei Li [1,]* and Honglei Zhang [1,]*

[1] State Key Laboratory of New Pharmaceutical Preparations and Excipients, Key Laboratory of Medicinal Chemistry and Molecular Diagnosis of the Ministry of Education, Key Laboratory of Chemical Biology of Hebei Province, College of Chemistry and Materials Science, Hebei University, Baoding 071002, China; yingyang@stumail.hbu.edu.cn (S.W.); persistent_ghw@stumail.hbu.edu.cn (H.L.); 2024400355@buct.edu.cn (X.F.); fushuangqing@stumail.hbu.edu.cn (S.F.)

[2] Department of Life Science, Hengshui University, Hengshui 053000, China; chaozhang@hsnc.edu.cn

* Correspondence: liwei2020@hbu.edu.cn (W.L.); zhanghonglei@hbu.edu.cn (H.Z.)

Supplementary Materials:

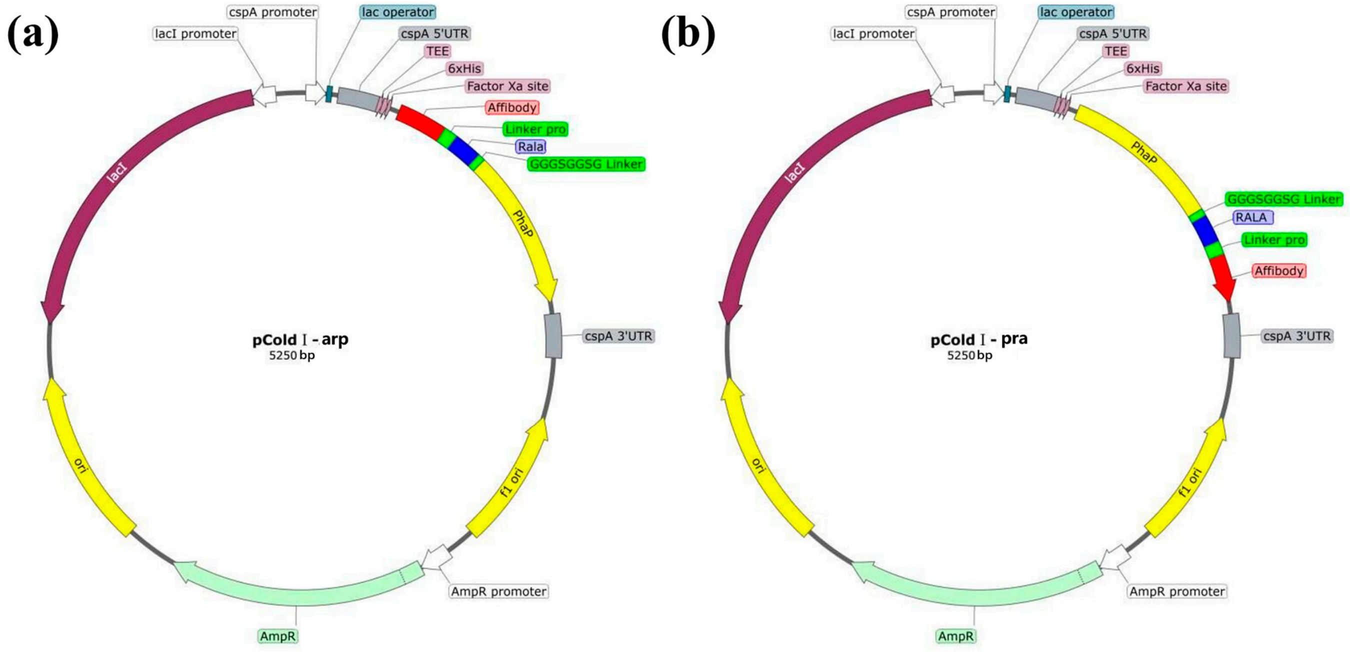

Figure S1. Expression plasmids containing two fusion protein genes of different orders. (a) pCold I-*affibody-rala-pha*P (pCold I-*arp*). (b) pCold I-*pha*P-*rala-affibody* (pCold I-*pra*).

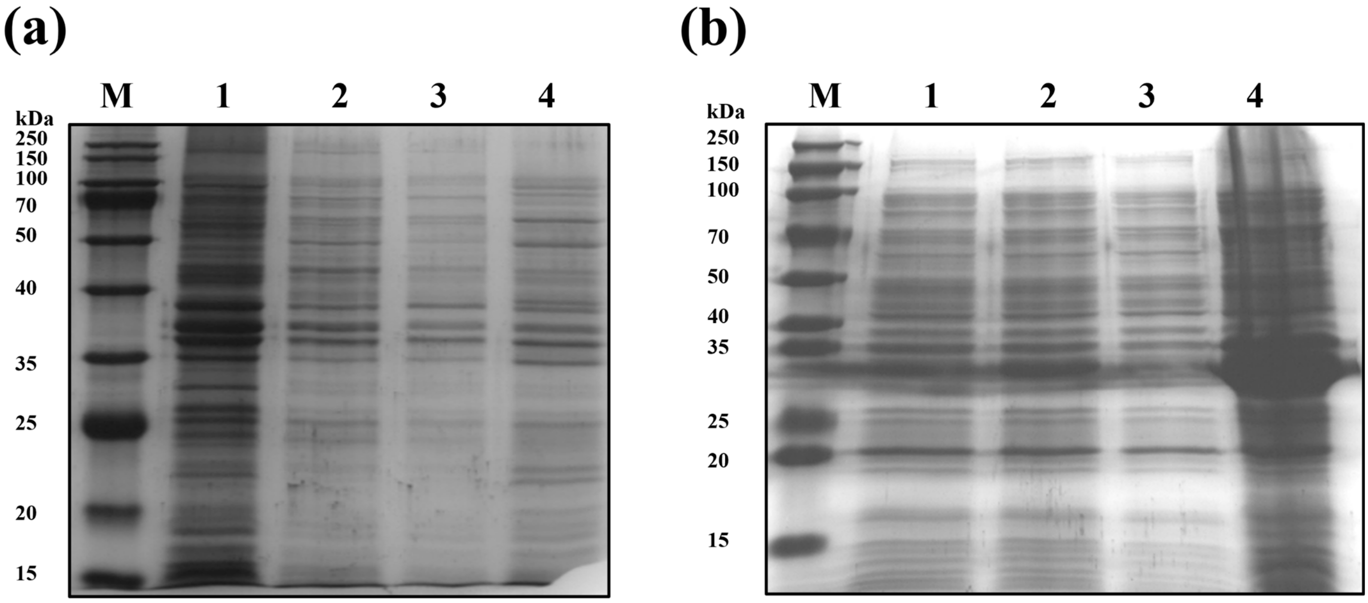

Figure S2. (a) Analysis of PRA protein by SDS-PAGE gel electrophoresis. (M: Marker; Lane 1: before IPTG induction; Lane 2: after IPTG induction; Lane 3: supernatant after crushing; Lane 4: bacterial precipitation after crushing). (b) Analysis of ARP proteins by SDS-PAGE gel electrophoresis. (Lane M: Marker; Lane 1: supernatant of crushed cells; Lane 2: after induction of ARP protein; Lane 3: before induction of ARP protein; Lane 4: bacteria precipitate after fragmentation).

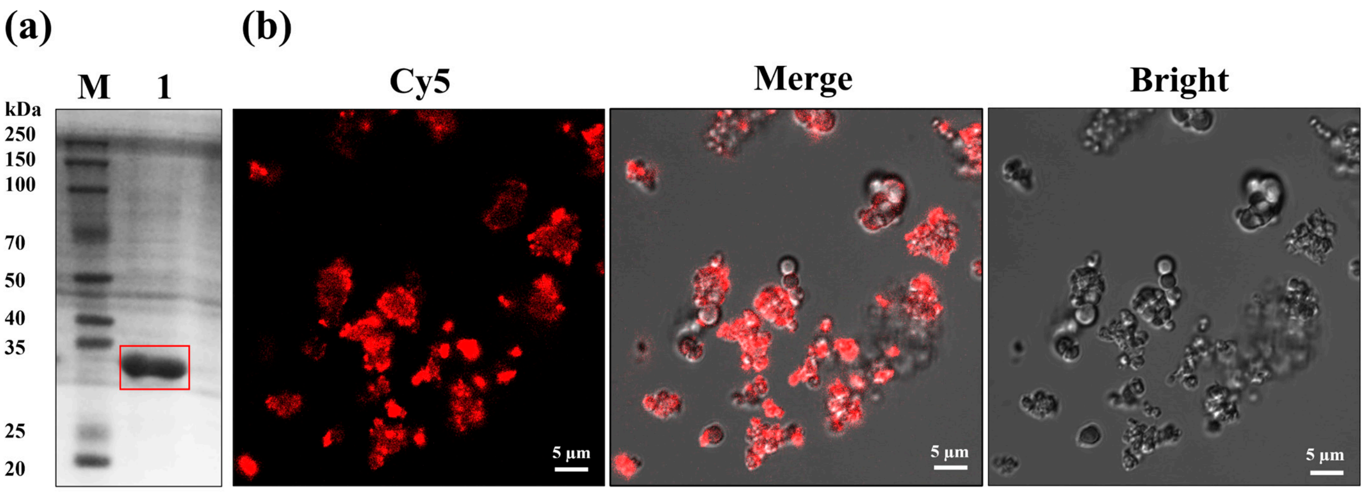

Figure S3. Verification of the correctly assembled triple-drug microspheres. (a) SDS analysis of the ARP protein was attached to the surface of the microspheres. (b) Confocal imaging validation of siRNA$_{GEM}$ was absorbed by microspheres.

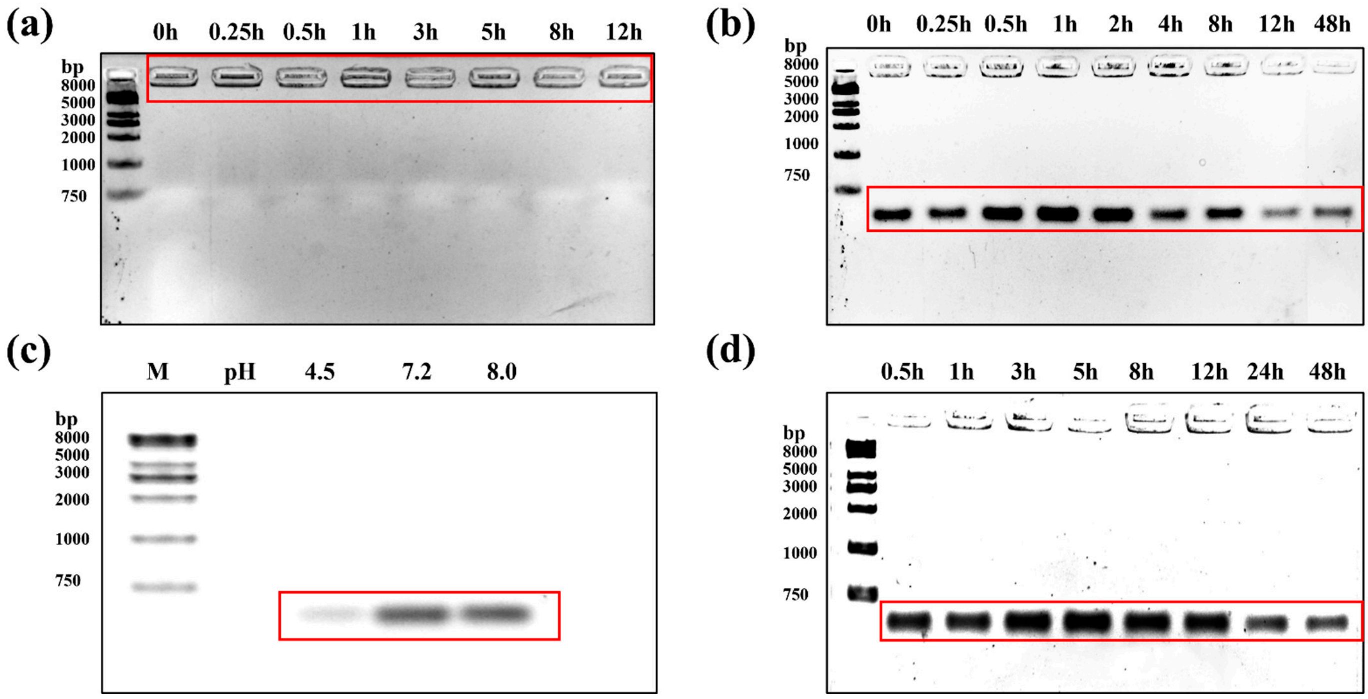

Figure S4. Analysis of the enzymatic release of PTX@PHBHHx-ARP/siRNA$_{GEM}$ by agarose gel electrophoresis. (a) The stability of PTX@PHBHHx-ARP/siRNA$_{GEM}$ at different times in 10% serum without heparin sodium treatment. (b) The stability of siRNA$_{GEM}$ on PTX@PHBHHx-ARP/siRNA$_{GEM}$ at different times in 10% serum with heparin sodium treatment. (c) The stability of PTX@PHBHHx-ARP/siRNA$_{GEM}$ at different pH. (d) The stability of PTX@PHBHHx-ARP/siRNA$_{GEM}$ in RNase I.

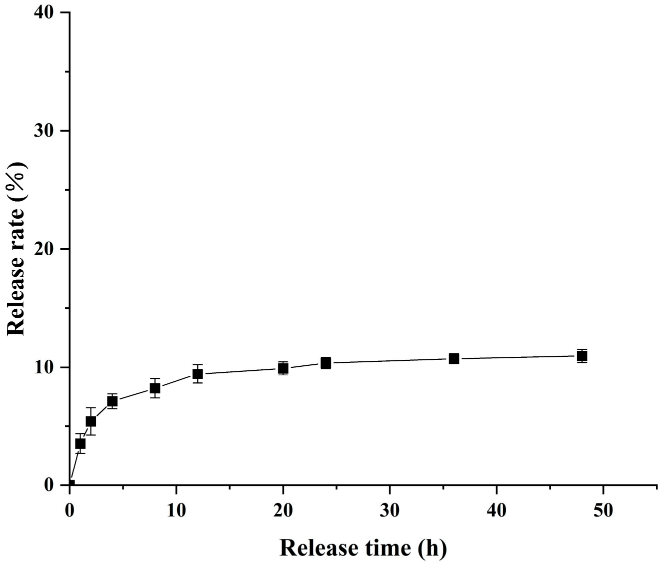

Figure S5. Kinetics of PTX release under neutral conditions.

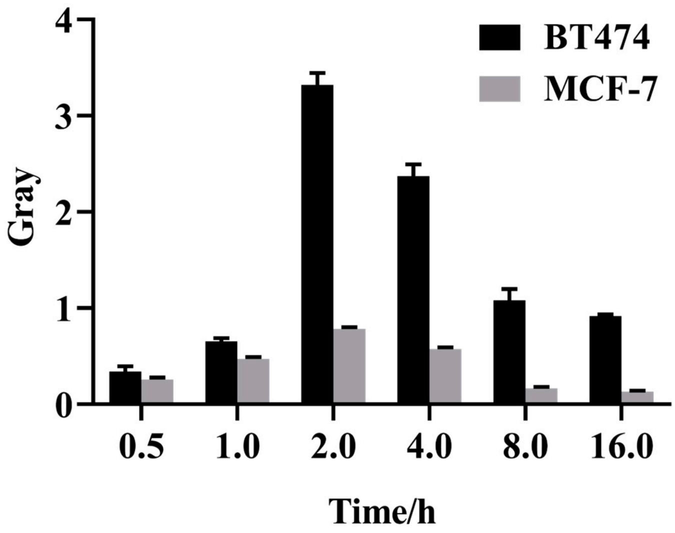

Figure S6. Grayscale control of uptake of PTX@PHBHx-ARP/siRNA$_{GEM}$ by two different cells.

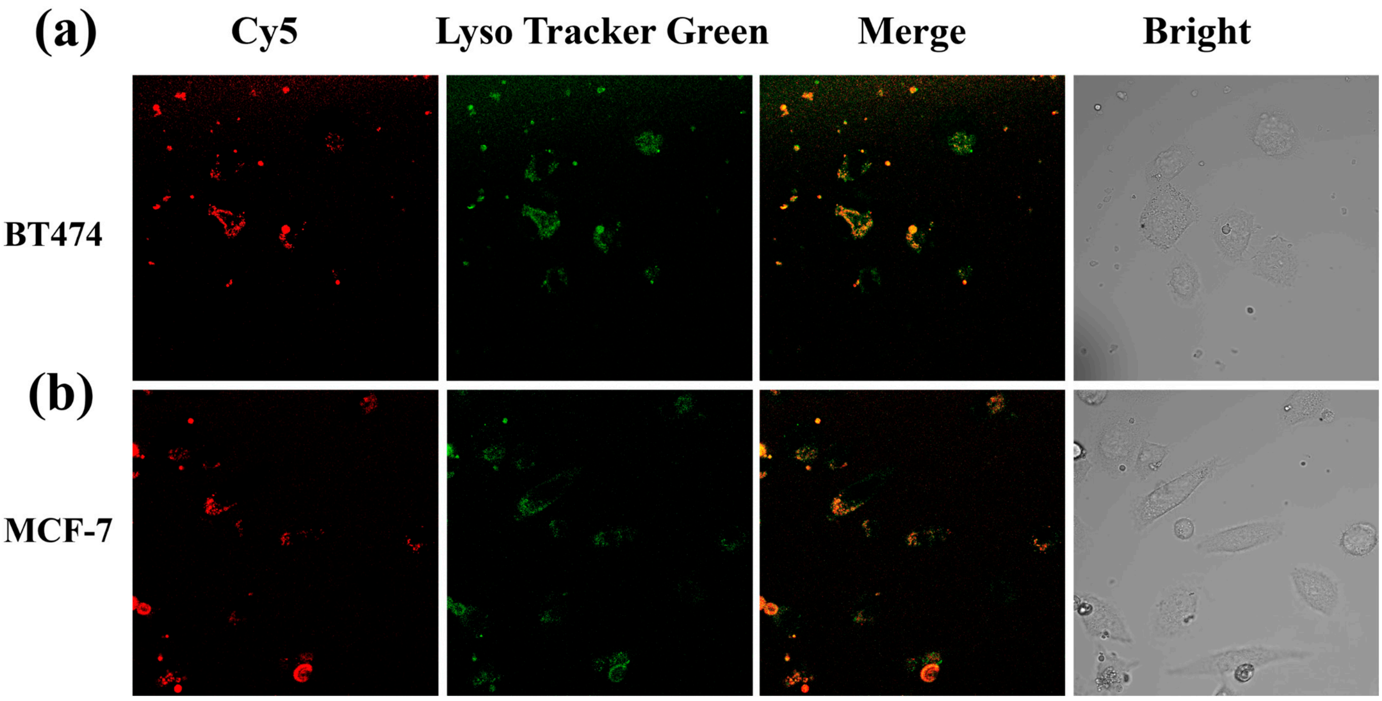

Figure S7. Intracellular localization map of the drug after uptake by BT474 (a) and MCF-7 (b) cells. After the cells were incubated with the drug for 2 h, the position of PTX@PHBHHx-ARP/siRNA$_{GEM}$ after its entry into the cells was observed by CLSM. Scale bar: 20 μm.

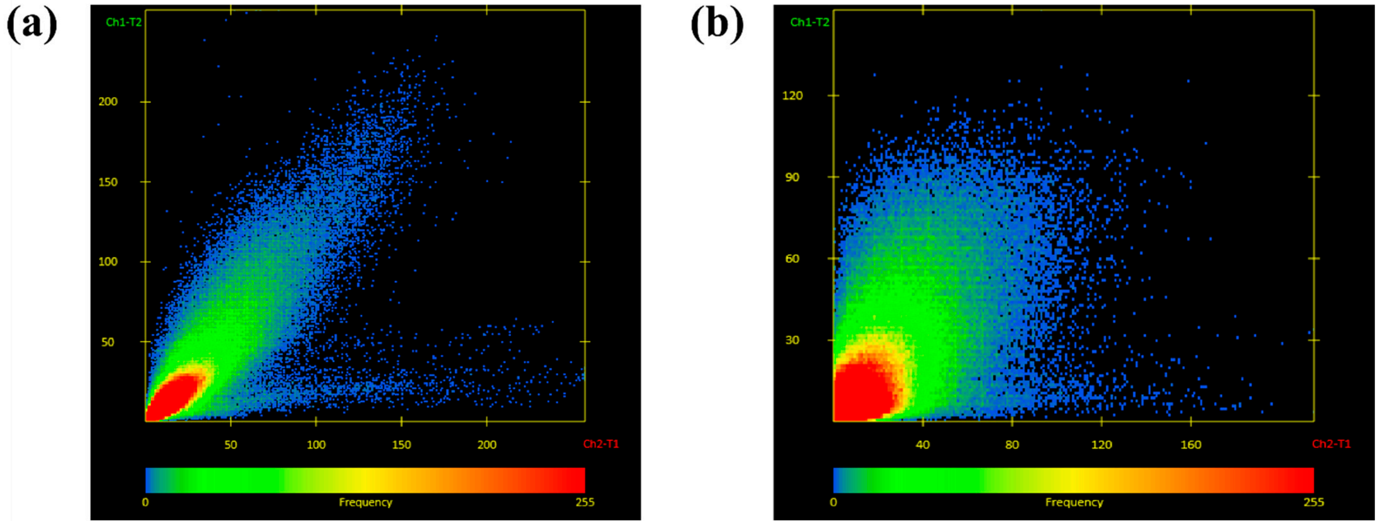

Figure S8. The co-localization of PTX@PHBHx-ARP/siRNAGEM in lysosomes was analyzed by software. (a) The co-localization of PTX@PHBHx-ARP/siRNAGEM in MCF-7 cells. (b) The co-localization of PTX@PHBHx-ARP/siRNAGEM in BT474 cells.

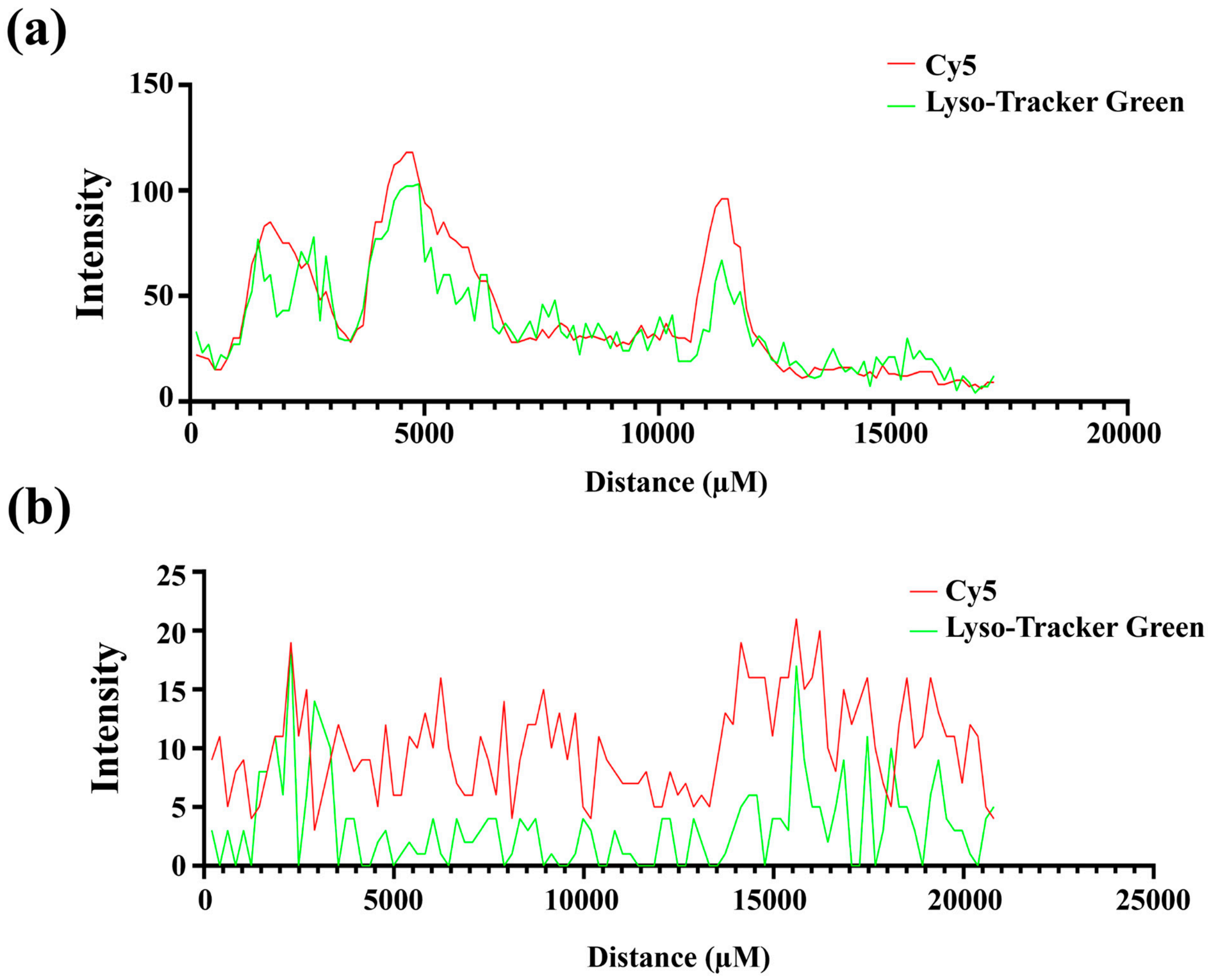

Figure S9. Localization analysis of PTX@PHBHx-ARP/siRNAGEM in lysosomes in both cell types. (a) Lysosomal co-localization in BT474 cells. (b) Lysosomal co-localization in MCF-7 cells.

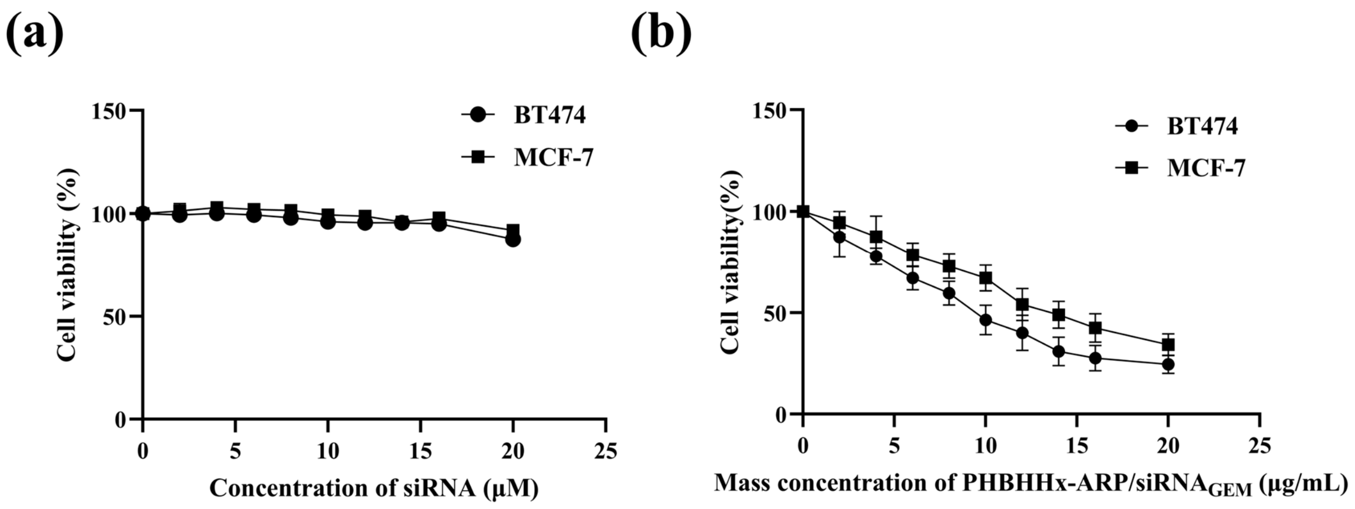

Figure S10. The survival rates of the two cell lines were observed after 48 h of treatment. (a) siRNA. (b) PHBHHx-ARP/$siRNA_{GEM}$.

Table S1

CI values of different cells treated with three different combinations of drugs

Cell line	CI	
	PTX/GEM/siRNA(1:9:170)	PTX@PHBHHx-ARP/$siRNA_{GEM}$
BT474	0.059	0.0007
MCF-7	0.150	0.0014
